# The effect of three violent videogame engagement states on aggressive behavior: A partial least squares structural equation modeling approach

**DOI:** 10.3389/fpsyg.2022.918968

**Published:** 2022-10-10

**Authors:** Amir Zaib Abbasi, Umair Rehman, Khalil Hussain, Ding Hooi Ting, Helmut Hlavacs, Hamza Qummar

**Affiliations:** ^1^IRC for Finance and Digital Economy, King Fahd University of Petroleum and Minerals, Dhahran, Ash Sharqiyah, Saudi Arabia; ^2^User Experience Design, Wilfrid Laurier University, Waterloo, ON, Canada; ^3^School of Hospitality and Service Management, Sunway University, Petaling Jaya, Malaysia; ^4^Department of Management and Humanities, Universiti Teknologi PETRONAS, Seri Iskandar, Malaysia; ^5^Entertainment Computing, University of Vienna, Vienna, Austria; ^6^Department of Management Sciences, Shaheed Zulfiqar Ali Bhutto Institute of Science and Technology, Islamabad, Pakistan

**Keywords:** consumer violent videogame engagement, cognitive, affective, behavioral engagement, aggressive behavior, reflective-formative, hierarchical component model

## Abstract

Debate on violent games and their effect on aggressive behavior remains inconclusive. This study aims to study the predicting role of cognitive, affective, and behavioral engagement states in violent videogames on aggressive behavior, which remains nebulous to date. We visited gaming zones and administered the study survey to collect data from violent videogame users. We collected 208 valid responses that were further analyzed. The present study used SmartPLS (3.3.3) software to perform partial least squares structural equation modeling (PLS-SEM) analysis in two stages. In the first stage, the measurement model assessment reported that cognitive, affective, behavioral, and aggressive behavior proved to be reliable reflective-formative composite constructs. Whereas, the second phase illustrated that cognitive engagement in violent videogames fails to impact aggressive behavior. The other two engagement states (affective and behavioral) in violent games showed a positive impact on aggressive behavior. Our study contributes to aggressive behavior literature by understanding how violent videogame engagement states impact aggressive behavior, which is crucial to recognize aggression so that steps can be taken toward addressing it. This study also contributes methodologically by utilizing the hierarchical component model (HCM) approach to estimate, specify, and validate the hierarchical structure of higher-order constructs (i.e., consumer violent videogame engagement dimensions (cognitive, affective, and behavioral) and aggressive behavior) as reflective-formative composite models.

## Introduction

With continuous technological innovations and development, video gaming has grown to become a global industry worth over a billion dollars and has unprecedented growth of $60.4 billion in 2022 from $43.4 billion in 2019 (Pierre-Louis, 2022).The industry continues to attract new customers with innovative new releases and developments every year (Cheng et al., 2015). One of the most popular genres of videogames that have been and continue to grow is the shooter games. The videogames have been designed that come with exaggerated displays of violence and adult imagery (Greitemeyer and Mügge, 2014). In previous meta-analyses, it has been suggested that violent videogames can increase aggressive behavior among youth (Przybylski and Weinstein, 2019; Burkhardt and Lenhard, 2022). However, this conclusion is not widely accepted and several studies have been conducted to test these. Through literature research, we found evidence of both positive and negative outcomes associated with videogame play under specific conditions and believe the findings should be considered in any future studies related to videogame violence (Przybylski and Weinstein, 2019). There is research that shows that exposure to violent videogames of competitive nature increased aggressiveness amongst players and this could be attributed to game engagement-related factors (Hawk and Ridge, 2021). This behavior is also evident in Greitemeyer and Mügge (2014), who believe that the popularity of violent videogames contributes to increased displays of public aggression. A report issued by the American Psychological Association (2015) agrees with the idea that consistent exposure to such games can be the cause of aggressive behavior, especially in teenagers. However, academic sources are divided over the matter.

In academic literature, there are three different notions of violent games and aggressive behavior. On the first note, many academic literatures postulate that increased displays of violence in videogames have a direct positive relationship with rising human aggression (Hollingdale and Greitemeyer, 2014; Riva et al., 2017; Greitemeyer, 2018; Tian et al., 2020). On the other hand, many academics argue that these studies are limited as they use self-reported data (Prot et al., 2014). Thus, self-reported data may influence the results, resulting in skewed and inaccurate conclusions. To overcome this issue, our study examined the health effects of violent videogames, i.e., how violence affects consumers’ well-being by causing potential health problems such as increased aggression and depression. The findings of our study are hoped to help in providing actions/interventions that can alleviate these negative effects (Kühn et al., 2019; Ferguson et al., 2020). Till today, the debate on videogame and aggressive behavior remains controversial with no hypothesis being accepted as universally conclusive (Markey et al., 2015). The most popularly used theoretical framework to investigate the association between violent videogame engagement and aggression is the well-known general aggression model (GAM). The debate has resulted in the formation of two schools of thought. One argues that engagement with violent games as studied through the GAM tends to increase aggression in the consumer’s behavior by desensitizing them to display violence and gore (Hollingdale and Greitemeyer, 2014; Tian et al., 2020). The extent of the link between media violence and aggression is still debated, but many studies provide evidence that engagement with violence in videogames has a negligible effect on the psyche of consumers (Przybylski and Weinstein, 2019; Johannes et al., 2022). Previous studies have reported that violent videogames amplify aggressive cognition, however, the relationship of aggressive cognition with aggressive behavior or hostile effect remained unclear (Drummond et al., 2021).

The first school of thought argues that aggression stemming from exposure to violent games can take numerous forms. Some of the most common ones are physical aggression with the intent of causing physical harm (punching, slapping etc.) and verbal aggression including but not limited to the use of abusive language, screaming, and passing derogatory remarks (Albina et al., 2020). These studies could be limited in their assessment due to the use of longitudinal data to analyze the community-level effect instead of individual data (Beerthuizen et al., 2017). The conclusions regarding the relationship between violent videogames and aggressive behavior have often been deemed inconclusive (e.g., see a review study by Drummond et al., 2020 and Ferguson and Wang, 2021). It remains unclear if exposure to violent videogames results in the growth of aggressive tendencies in consumers (Romanchych, 2018).

Many studies have defined and discussed the impact of violence in videogames (Hollingdale and Greitemeyer, 2014; Ferguson and Wang, 2019, 2021; Przybylski and Weinstein, 2019; Denson et al., 2020; Ferguson, 2020; Ruiz-Fernández et al., 2021). Bassiouni and Hackley (2016) state that videogames may have potentially negative effects on children’s sense of identity. In contrast, based on self-reported data evidence from the register report, Przybylski and Weinstein (2019) concluded that violent videogame engagement is not related to teenagers’ aggressive behavior. Przybylski et al. (2010) provided a motivational model of videogame engagement that is originated from self-determination theory (SDT) to predict the motivational sources (e.g., need for competence, autonomy, and relatedness) of post-play aggression. Surprisingly, none of them has brought up the kind of exposure that propagates feelings of aggression among the consumers after being engaged in violent videogames. Therefore, it is important to understand the degree of virtual involvement that affects the users’ real-life aggression (Hollebeek, 2013; Hollebeek et al., 2020). This motivates us to investigate these phenomena, with a particular focus on gamers’ engagement states comprising cognitive, behavioral, and affective that ignite the consumers’ aggression through continued exposure to violent videogames.

This study contributes to the existing knowledge by providing a deeper understanding of gamers’ aggressive behavior and GAM on how gamers’ violent videogame engagement states (affective, behavioral, and cognitive) contribute to explaining the gamers’ aggressive behavior. We outline our research questions based on this debate:

1. Does cognitive engagement influence aggressive behavior?
2. Does affective engagement influence aggressive behavior?
3. Does behavioral engagement influence aggressive behavior?

## Literature review

Our research model has predictors that include cognitive engagement, affective engagement, and behavioral engagement that may lead to the gamers acting aggressively. In this study, aggressive behavior has been taken as the consequence of cognitive, affective, and behavioral engagements.

### Cognitive engagement

Cognitive engagement refers to engaging consumers in effortful tasks that entail determination and strategy (Ge and Ifenthaler, 2018; Abbasi, 2022). These usually include a set of activities that give the user a feeling of being involved (conscious attention) in the subject matter. This form of engagement attracts the user, even more, allowing them to truly be immersed in the gaming experience (Higgins and Scholer, 2009). Conscious attention is similar to the dimension of immersion and can be defined and measured as the interest shown by a person (Vivek et al., 2014). Additionally, absorption is a high level of concentration and engrossment that includes a loss of self-consciousness, a lack of appreciation for the time, and inborn satisfaction (So et al., 2016). Cognitive engagements can involve immersive forms of role-playing or pretend play (Wu and Holsapple, 2014), as well as situations involving intense problem solving (Tsang et al., 2012). Given the immersive effects of engagement, players who lose while playing these games might showcase increased aggression in their behavior as a consequence of increased frustration (Griffiths et al., 2016). These engagements may bring hostile attribution bias (such as a change in the users’ attitude towards violence) that seems to affect and challenge stable thoughts and beliefs that develop over a lifetime (Anderson et al., 2010). GAM agrees with the notion that high attention to violent videogames increases consumers’ aggressive thoughts, and hostile expectations, and lowered their level of tolerance; especially when playing with human opponents (Hollingdale and Greitemeyer, 2014). This study focuses on cognitive engagement that leads to aggressive behavior through violent videogames. Based on the discussion, it is purposed that:

*H1:* Cognitive engagement with violent videogames has a positive relationship with aggressive behavior in consumers.

### Affective engagement

Affective engagements refer to a form of situational interest (Parsons et al., 2012). It is defined as the summative and enduring level of emotions and it involves the consumer showcasing increased amounts of enthusiasm and dedication (Abbasi et al., 2019b, 2021a). Enthusiasm can be measured through the positive emotions displayed by a consumer while interacting with a product with complete focus (Vivek et al., 2014). On the other hand, dedication is used to express a sense of belonging to an artifact (Schaufeli et al., 2002). With such levels of engagement, we believe that most videogame players showcase a perceived emotional state corresponding to the situation on screen. When these users face hostile situations in violent videogames, they often display acts of aggression to exercise their perceived emotions (Triberti et al., 2015). Violent displays in videogames stimulate regions of the brain known to be affected by anger (Tear and Nielsen, 2013). Consequently, affective engagement can lead to real life displays of physical aggression and violence (Anderson et al., 2008). Based on this discussion, this study proposes that:

*H2:* Affective engagement with violent videogames is positively associated with aggressive behavior.

### Behavioral engagement

**Figure 1:**
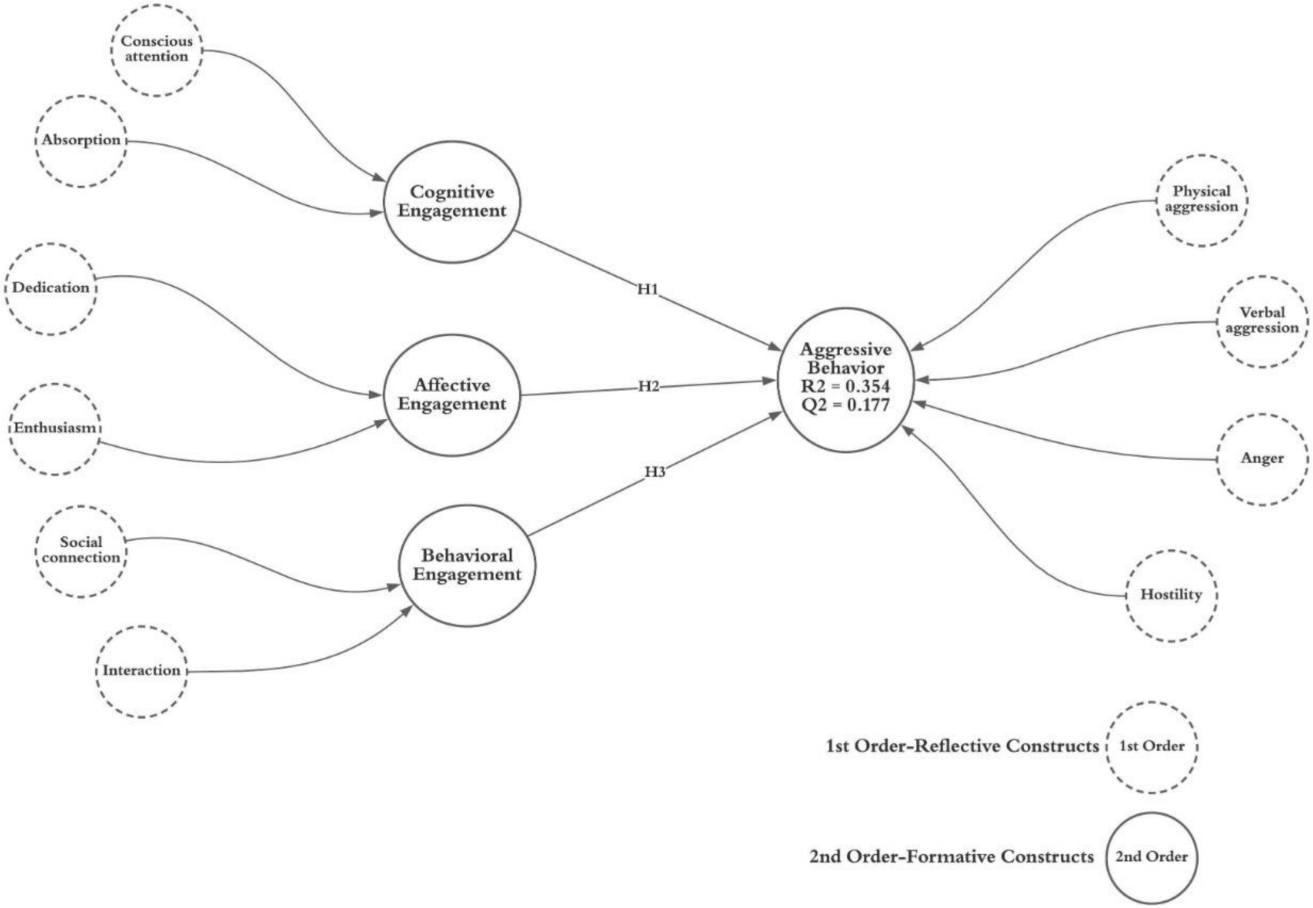
A conceptual model.

Behavioral engagement refers to observable actions showing signs of attention, participation, and involvement of a user (Ge and Ifenthaler, 2018; Shah, 2019). Behavioral engagement is evident from the behavioral manifestation, largely due to certain aspects of the game such as social connection and interaction (Abbasi et al., 2019b). Interaction involves sharing and exchanging ideas, thoughts, and feelings concerning an experience (So et al., 2016). Social interaction relies on the involvement of others while the attentions of engagement depict the combined action with other members of society (Vivek et al., 2014). With regard to violent videogames, whenever a player perceives a certain difficulty which hinders their enjoyment and engagement, it increases their tendency to be aggressive (Anderson et al., 2004). While the violence in the game can be in the form of animosity or a composite of physical and verbal aggression (Anderson et al., 2010), it is a significant causal factor for physically aggressive behavior in its consumers (Anderson et al., 2008). In other words, we have good reason to believe that when a gamer is exposed to violent videogame engagement, the exposure and learning processes (and through the priming of aggressive thoughts) might be associated with human aggression. Based on the model as depicted in Figure 1, this study proposes that:

*H3:* Behavioral engagement with violent videogames is positively associated with aggressive behavior.

## Methodology

### Sample and data collection

In this study, we used the cross-sectional survey design due to its effectiveness in getting faster responses (Creswell, 2013). Our study data were collected in two main cities of Pakistan; Islamabad and Rawalpindi. Both cities are high metropolitan areas and are greatly contributing to the Pakistan economy. Both cities have different gaming zones and consumers. In many instances, the young generation has the highest tendency to continuously engage in video gaming (Abbasi et al., 2020). Thus, our study involved a generation Z (8–24 years old) group who play violent videogames categories [e.g. first-person shooter (FPS), real-time strategy (RTS), multiplayer online battle arena (MOBA), and role-playing game (RPG)]. We choose generation Z because generation Z considers the effective use of digital technology, e.g., videogames (Emre, 2020). The prior study illustrates that generation Z is considered a prospective subject for examination in digital videogames (Bassiouni and Hackley, 2014). To reduce the methodological sampling bias, we use judgmental sampling. It is a feasible sampling procedure for accumulating data from a few categories of gamers. Here, we visited different gaming zones (e.g., Safa Gold Mall VR Fun Zone, Fun City Centaurus Mall, Epic Gaming Zone, Galaxy Gaming Zone, and Vortex Reborn Gaming Lounge). We have obtained permission from the gaming zone representative to approach our respondents. We collected data from these game zones after players finished their games (exited the gaming centers).

To determine the required samples, we used the G∗power 3.0 (Faul et al., 2007). The following commands were given, we input *F-tests* family, statistical test (*linear multiple regression: fixed model, R^2^ deviation from zero*), effect size, *f*^2^ = 0.15, α err prob. (0.05), power (0.95), and several predictors = 3. G*power 3.1.9.2 acclaims a sample size of 119 for the current study model. 150 sample size is an acceptable parameter for measuring fewer than seven constructs (Hair et al., 2010; Abrahim et al., 2019). Based on the power analysis, we aimed to collect around 250 to increase the generalizability of the study results. However, we only managed to collect 230 questionnaire data from respondents in Islamabad and Rawalpindi. Out of the collected data (i.e., 230 responses), 22 were removed due to erroneous results (e.g., we found likenesses in the responses; too many neutral answers and some of the replies followed an odd answer pattern). Table 1 presents an outline of the participants’ demographics. Out of 208 respondents, 123 (59.1%) were male and 85 (40.9%) were female. Similarly, the education level is as follow: 133 (63.9%) bachelor, 54 (26%) intermediate, 18 (8.7%) Master, and 3 (1.4%) matric students. The distribution of the game categories is; FPS, battlefield 13.5%, RTS Age of empire 26.4%, MOBA online battle arena 51.9%, and RPG witcher series 8.2%.

**Table 1:** Overview of respondents’ demographics.

Respondents’ demographic	Frequency	Percentage (%)
Total	208	100
Gender		
Female	85	40.9
Male	123	59.1
Age		
16–18	5	5
19–20	127	127
21–22	60	60
23–24	16	16
Education		
Matric	3	1.4
Intermediate	54	26.0
Bachelor	133	63.9
Master	18	8.7
PHD	0	0
Games categories		
FPS, battlefield	28	13.5
RTS Age of empire	55	26.4
MOBA online battle arena	108	51.9
RPG witcher series	17	8.2

We employed a previously validated instrument to assess videogame engagement (Abbasi et al., 2016, 2017) that has been validated across several studies, e.g., consumer eSports videogame engagement (Abbasi et al., 2020, 2021a), serious game engagement (Abbasi et al., 2022), and in general video gaming contexts (Abbasi et al., 2019a, 2021b). The scale comprised video engagement dimensions, including, cognitive engagement factors, such as conscious attention and absorption; affective engagement factors, such as dedication and enthusiasm; and behavioral engagement factors, such as social connection and interaction. The scale that we adapted to assess aggressive behavior was also a standardized scale previously validated. It is comprised of factors such as physical aggression, verbal aggression, anger, and hostility (Buss and Perry, 1992). Five-point Likert scales (1: strongly disagree, 2: disagree, 3: neutral, 4: agree, and 5: strongly agree) were used. The questionnaires were divided into two sections; section 1 is on the demographics of the digital videogamers (e.g., game categories, age, gender, and qualification). Section 2 involved the violent videogame engagement states comprising cognitive engagement, affective engagement, and behavioral engagement and aggressive behavior, see Appendix A for detailed information on the measurement items.

### Data analysis process

The research framework illustrated in Figure 1 is analyzed using partial least squares structural equation modeling (PLS-SEM). It is a promising technique when dissimilar variables are analyzed together and the research objective is testing new relationships as well as theory building (Hair et al., 2011, 2020). For the present study, we applied the PLS-SEM approach because the variables involved in the present study have both reflective and formative constructs (Hair et al., 2019). Thus, we used Smart_PLS 3.2.8 to perform the analysis (Hair et al., 2020). The analysis is divided into two stages; measurement model analysis and structural model analysis.

## Results

### Assessment of the measurement model

As illustrated in Figure 1, our study proposes a model that includes different constructs of consumer videogame engagement and aggressive behavior. First, consumers’ violent videogame engagement is a multidimensional construct consisting of three second-order formative constructs, e.g., behavioral, affective, and cognitive engagement (Abbasi et al., 2019a). In the present study, violent videogame engagement constructs are: cognitive engagement–quantified by absorption and conscious attention, affective engagement is specified *via* dedication and enthusiasm and finally behavioral engagement is assessed through social connection and interaction. Second, aggressive behavior is elucidated in the second-order formative construct which categories are verbal aggression, physical aggression, anger, and hostility.

### Reflective model assessment

To measure the reflective constructs, we test the reliability and validity of the scales. The outer loading, Cronbach’s alpha and composite reliability (CR) were used to test the reliability of the corresponding measurement models. The average variance extracted (AVE) was used to test the convergent validity. The Heterotrait-Monotrait Ratio (HTMT) ratio of correlation was used to test the discriminant validity. In our study, except for some items (e.g., ANG2, 4 and 5; HOS7, DED1 and 3; ENT6, PAG3, 5, 8 and 9), all outer loadings are greater than the threshold of 0.6, these items were loaded within 0.3 and 0.59 and this would be retained if the AVE and CR are greater than the benchmark. Cronbach’s alpha and CR should surpass 0.70 to ensure internal consistency. In the present study, all construct values are higher than the thresholds. If the value of AVE is greater than 0.5, then convergent validity is assured. Again, all dimensions surpass the AVE threshold, thus convergent validity is established. Table 2 reports the corresponding results.

**Table 2:** Results of the assessment of the measurement model reflective constructs.

Constructs	Items	Loadings	Cronbach’s Alpha	CR	AVE
Conscious Attention	CA1	0.706	0.848	0.885	0.562
	CA2	0.712			
	CA3	0.786			
	CA4	0.766			
	CA5	0.737			
	CA6	0.788			
Absorption	ABS1	0.787	0.825	0.877	0.588
	ABS2	0.738			
	ABS3	0.806			
	ABS4	0.781			
	ABS5	0.717			
Dedication	DED1	0.678	0.789	0.856	0.544
	DED2	0.773			
	DED3	0.686			
	DED4	0.742			
	DED5	0.800			
Enthusiasm	ENT1	0.724	0.780	0.850	0.532
	ENT2	0.761			
	ENT3	0.704			
	ENT4	0.760			
	ENT5	0.696			
Social Connection	SOC1	0.854	0.795	0.878	0.706
	SOC2	0.845			
	SOC3	0.821			
Interaction	INT1	0.825	0.865	0.902	0.647
	INT2	0.782			
	INT3	0.826			
	INT4	0.791			
	INT5	0.796			
Physical Aggression	PAG1	0.784	0.863	0.892	0.510
	PAG2	0.761			
	PAG3	0.639			
	PAG4	0.791			
	PAG5	0.582			
	PAG6	0.788			
	PAG8	0.667			
	PAG9	0.666			
Verbal Aggression	VA1	0.757	0.771	0.852	0.590
	VA2	0.770			
	VA3	0.708			
	VA4	0.831			
Anger	ANG2	0.554	0.821	0.869	0.531
	ANG3	0.860			
	ANG4	0.593			
	ANG5	0.760			
	ANG6	0.784			
	ANG7	0.769			
Hostile	HOS1	0.734	0.784	0.852	0.537
	HOS2	0.778			
	HOS3	0.806			
	HOS4	0.713			
	HOS7	0.621			

The discriminant validity can be measured through a novel method—The Heterotrait-Monotrait (HTMT) ratio of correlations (Henseler et al., 2015). The HTMT ratio for all constructs should be less than the threshold value of 0.85. Table 3 exhibits the HTMT values indicating that all values are below 0.85, so the present study has no discriminant validity issue.

**Table 3:** Discriminant validity (HTMT) analysis.

	ABS	ANG	CA	DED	ENT	HOS	INT	PHY-AGG	SOC-CON	VER-AGG
ABS										
ANG	0.333									
CA	0.621	0.222								
DED	0.471	0.362	0.664							
ENT	0.679	0.414	0.469	0.722						
HOS	0.414	0.770	0.378	0.426	0.502					
INT	0.640	0.340	0.644	0.444	0.538	0.496				
PHY-AGG	0.536	0.708	0.413	0.435	0.436	0.711	0.519			
SOC-CON	0.645	0.155	0.521	0.370	0.549	0.437	0.717	0.484		
VER-AGG	0.270	0.676	0.279	0.257	0.258	0.513	0.444	0.588	0.348	

ABS, Absorption; ANG, Anger; CA, Conscious attention; DED, Dedication; ENT, Enthusiasm; HOS, Hostile; INT, Interaction; PHY-AGG, Physical aggression; SOC-CON, Social connection; and VER-AGG, verbal Aggression.

### Assessment of second-order formative constructs

To establish the second-order formative constructs in the present case; e.g., cognitive, behavioral, affective engagement, and aggressive behavior, Becker et al. (2012) suggest a two-step method. Firstly, we evaluate the latent-variable results of the first-order reflective constructs in the study. Secondly, we employ the achieved score of first-order reflective constricts as an indicator for modeling the second-order formative constructs. To test the validity of second-order formative constructs, first, we estimate the variance inflation factor (VIF), which is repeatedly used to assess the multicollinearity of the formative indicators. If the value of VIF is higher than the threshold of 5, that indicates collinearity issues among the indicators. We followed the measurement weight of the indicator and significant level to evaluate reliability and validity. Hence, in the present study, Table 4 exhibits all indicator weights for the second-order formative models, e.g., cognitive engagement, affective engagement, behavioral engagement, and aggressive behavior are significant that indicate reliable and valid second-order formative constricts.

**Table 4:** Validity tests second-order constructs.

Second order formative construct	Items	Outer-weights	Standard deviation (STDEV)	T Statistics (|O/STDEV|)	*p* values	VIF
Cognitive Engagement	Absorption → Cognitive Engagement	0.599	0.129	4.646	**0.000** ^***^	1.374
	Conscious Attention → Cognitive Engagement	0.547	0.133	4.130	**0.000** ^***^	1.374
Affective Engagement	Dedication → Affective Engagement	0.547	0.176	3.100	**0.001** ^***^	1.461
	Enthusiasm → Affective Engagement	0.585	0.167	3.491	**0.000** ^***^	1.461
Behavioral Engagement	Interaction → Behavioral Engagement	0.684	0.154	4.431	**0.000** ^***^	1.525
	Social Connection → Behavioral Engagement	0.432	0.168	2.563	**0.005** ^**^	1.525
Aggressive Behavior	Physical Aggression → Aggressive Behavior	0.667	0.148	4.510	**0.000** ^***^	1.98
	Verbal Aggression → Aggressive Behavior	0.295	0.146	2.017	**0.022** ^*^	1.503
	Anger → Aggressive Behavior	−0.394	0.195	2.020	**0.022** ^*^	2.253
	Hostile → Aggressive Behavior	0.527	0.162	3.260	**0.001** ^***^	2.041

Indicator weights significance at ***: *p* < 0.001;

** *p* < 0.01;

* *p* < 0.05.

### Assessment of the structural model

After the assessment of the measurement model, the structural model is required to check the relationship among latent variables. Thus, the values of coefficients of determination *R^2^* and Stone-Geisser Q2 were assessed and estimated on the bases of their threshold greater than zero. Table 5 illustrates that *R*^2^ and *Q*^2^ values have fulfilled the given criteria.

**Table 5:** Results of hypothesis testing.

Hypothesis	Original sample (O)	Sample mean (M)	Standard deviation (STDEV)	T Statistics (|O/STDEV|)	*p* values	*f* ^2^	*R* ^2^	*Q* ^2^
Cognitive Engagement → Aggressive Behavior	0.121	0.132	0.090	1.135	0.128	0.010	0.354	0.177
Affective Engagement → Aggressive Behavior	0.152	0.160	0.109	1.680	**0.046** ^*^	0.023		
Behavioral Engagement → Aggressive Behavior	0.411	0.412	0.106	3.756	**0.000** ^***^	0.140		

Significant at ***: *p* < 0.001;

** *p* < 0.01;

* *p* < 0.05.

**Figure 2:**
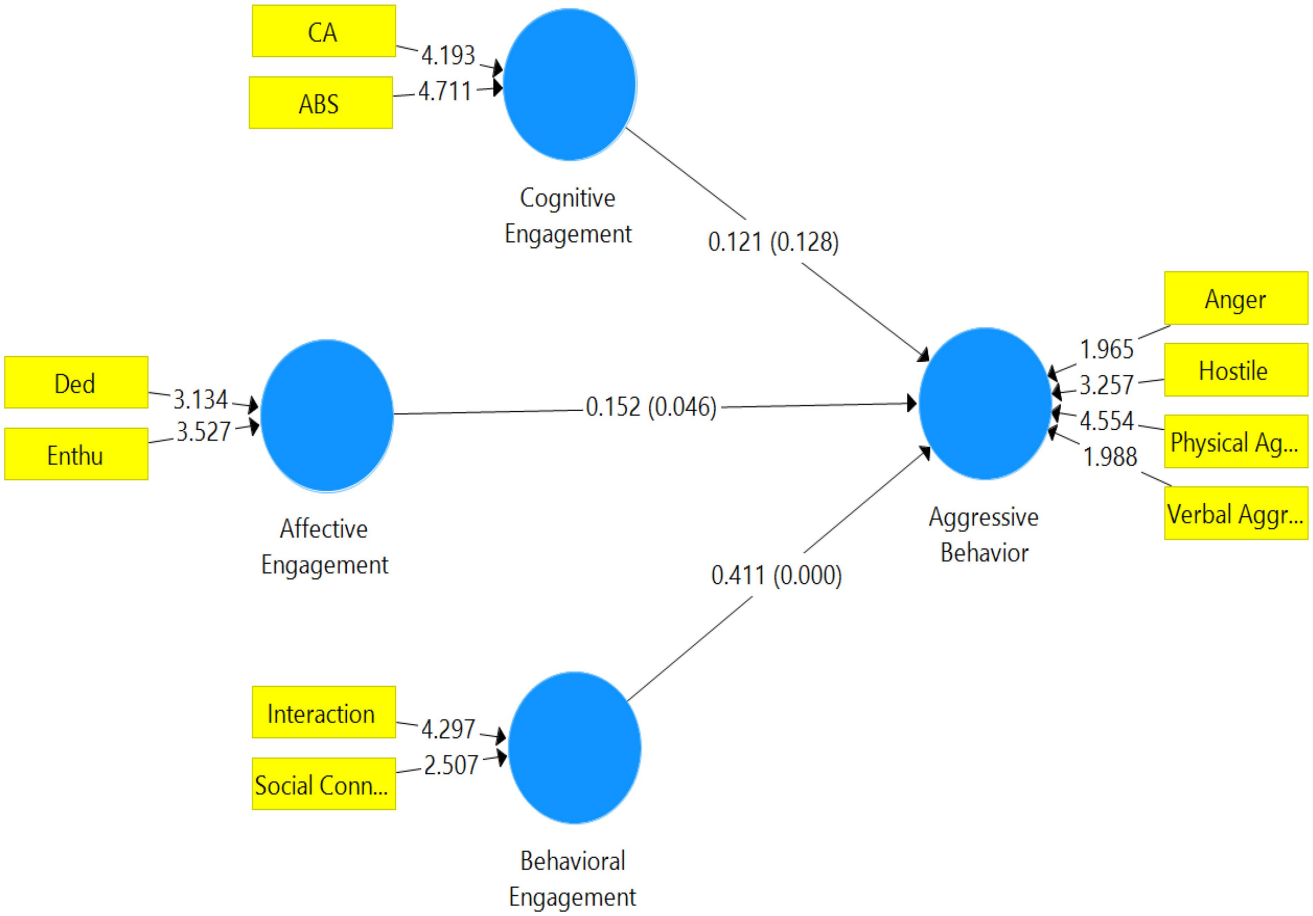
A conceptual model with hypothesis testing.

To test the hypotheses, we run the bootstrapping using Smart_PLS (v.3.2.8) with a subsample of 5,000 from the usable sample size of 208. Table 5 shows the path coefficient, mean, standard deviation and value of *p* for the corresponding paths. The results illustrate violent videogame cognitive engagement has an insignificant positive relationship with aggressive behavior, whereas, affective engagement and behavioral engagement have a significant positive relationship with aggressive behavior. Hence H1 is rejected, while H2 and H3 are accepted (Figure 2).

## Discussion

The study provides insights into the validity of the aggressive behavior prediction model through violent videogame engagement, which is very important in today’s world because of its effect on young violent videogamers (Romanchych, 2018). The subject of this study is highly relevant in this contemporary world where violent video gaming is no longer restricted to adult gamers. In fact, these games are now accessible to every consumer who has a digital device such as a smartphone, tablet, or laptop, which are commonly owned. This study provides further evidence that engagements play a significant role in determining the cause of aggression in consumer behavior.

Today, most of the games are available at very low prices or some are free of charge, hence, gamers from every age group have access to download and play the games. Most of these games have violent content and violent in nature games affect the aggressive outcomes. Few scholars have debated that violent videogames do have a substantial effect on aggressive behavior, especially among the childhood to early adolescence (Burkhardt and Lenhard, 2022). However, it may steadily decrease in the adulthood (Burkhardt and Lenhard, 2022). Hence, we conclude that the association between violent videogame engagement and aggression is still a concern and debatable area of research for many scholars due to having inconsistencies and contrary findings. For instance, prior studies reported that there is a negligible effect of violent videogames on aggression (Ferguson et al., 2020; Johannes et al., 2022) and while others have supported this association (Zhang et al., 2021a). In this scenario, the present study developed and verified a framework that explores the relationship between violent videogame engagement and aggressive behavior. This is because, numerous studies have assessed and defined the impact of violence in videogames on the psyche of consumers, but virtually none of them have depicted the three levels of engagement (e.g., cognitive, affective, and behavioral) that bring out the aggression in the behavior of the users while playing the said videogames. This study found that in the forms of engagement when participants are immersed in a competitive gaming environment, the aggression level in their behavior increases even further. In addition, it can be argued that a combination of these engagements including cognitive engagement, affective engagement, and behavioral engagement is the cause of such behavior.

In the following part of the paper, we discuss the prominent findings from our empirical study. The study supported the arguments of many academics who believe that being immersed in the competitive videogame environment brings out aggressive tendencies in the players’ behavior and losing these games increases such aggression (Adachi and Willoughby, 2011, 2016; Breuer et al., 2015; Griffiths et al., 2016). The results indicate that violent videogame engagement resulted in aggressive behavior in generation Z gamers. However, cognitive engagement failed to impact the players’ behavior, which is in line with previous research and arguments that users are less cognitively-driven when engaged with violent videogames (Drummond et al., 2021). A common reason for this is that the environment itself helps shape the user’s cognition and sets up the behavioral models. Moreover, the content of the game play also has an important role since it is the content that holds the power to cognitively drive the young players or not. Integrating this finding into an empirical conclusion, it is safe to say that the development of affective (emotional) engagement and behavioral engagement with violent videogames provide a way to comprehend the increased aggressiveness of the players because current study finding illustrates behavioral engagement has a highly significant effect on aggressive behavior whereas affective engagement has a partial impact on aggressive behavior. This finding is in line with past studies (Romanchych, 2018; Hawk and Ridge, 2021).

### Theoretical implications

This study brings several theoretical contributions. First, we present an empirical study of consumer engagements and their effect on aggressive behavior, thereby extending the conceptual or exploratory findings of such scholars (Ferguson and Wang, 2019, 2021; Ruiz-Fernández et al., 2021; Zhang et al., 2021b) and adding insights into GAM. We expanded earlier research by investigating the impact of cognitive, affective, and behavioral engagements in violent videogames on gamers’ aggressive behavior. Our study findings bring new insights into the literature that affective and behavioral engagements with violent videogames were found to be the lead cause of fostering aggressive tendencies in consumer behavior.

Additionally, by looking into the effects of different levels of engagement with a videogame, the current study provides insight into GAM itself. It highlights how affective engagements and behavioral engagements are key drivers when it comes to behavioral fluctuations in videogame players. Many studies into the effects of violent videogames and their link with player aggression, for example, Tian et al. (2020) depict no significant impact on consumers’ behavior. On the other hand, the existing study has specifically examined the effect of different dimensions of videogame engagement on behavioral development, which is a novel contribution to this field of study. Besides, we have also provided a methodological contribution [i.e., employing the hierarchical component model (HCM) approach; Sarstedt et al., 2019], especially specifying, estimating, and validating the hierarchical structure of aggressive behavior and consumer engagement states comprising cognitive, affective, and behavioral as reflective-formative composite models in the context of violent games.

### Practical implications

This study has drawn several practical implications, including recommendations for game developers and marketers as well as scholars who can use this model to assess consumers’ engagement with games and its effects on their behavior. By scrutinizing the engagement factors, researchers can now predict the nature and extent of how people will be affected by exposure to violent videogames. This research also advances a viewpoint that can empower game developers to evaluate how a videogame stimulates the cognitive, affective, and behavioral-engagement states of a player. With this information in hand, game developers can adapt and tailor videogames to ensure that the engagement factors leading to aggressive behavior are tackled without compromising the positive effects such as gamers’ cognition, evaluation skills, measurement, and resilience in tasks (e.g., study, job, and daily chore). By employing our model, game developers can strategically employ game effects (sounds, background music, animated characters, and visual effects) and game mechanics (e.g., rewards and playing strategies), which can lead to a reduction in aggressive tendencies. Overall, the present research provides evidence-backed findings that can be useful for the videogame industry as it can allow game developers to create violent videogames that do not lead to intense forms of aggressive behavior.

### Limitations and future direction

Despite having significant implications for the study, we have observed the following limitations. For instance, we only consider the age group that is only suitable for generation Z. Another study is much needed that could go beyond generation Z. Larger sample size would offer confirmatory evidence on research results and the current study must be conducted in other geographic locations to reveal additional nuances and variability in the current findings. Since the study respondents predominantly hailed from Islamabad and Rawalpindi regions, therefore, the current study findings may not apply to other regions where a change in culture and demographics may play a role in influencing current findings. In this study, we have observed that cognitive engagement in violent videogames fails to explain gamers’ aggressive behavior. The future study may incorporate the possible mediator to explain the unexplained relationship. Besides, personality traits may be investigated as possible moderators to determine what personality traits help in strengthening the relationship between violent videogame engagement states and gamers’ aggressive behavior. We limit our study to the consumption of violent videogames. However, future work can replicate the existing study model among generic videogame players to see whether engagement in generic videogame also influences gamers’ aggression or not. We also did not observe the effect of control variables (e.g., prior exposure to violence and amount of games being currently played) on determining gamers’ aggressive behavior. Thus, It is suggested that a future study should account for those control variables to explore whether such variables cause aggressive behavior or not.

## Data availability statement

The raw data supporting the conclusions of this article will be made available by the authors, without undue reservation.

## Author contributions

AA and UR worked on idea development and conceptualization. KH and HQ worked on literature and findings, which were further edited by HH and DT. KH helped in data collection. AA performed analyses. HH and DT also edited the whole draft. All authors contributed to the article and approved the submitted version.

## Conflict of interest

The authors declare that the research was conducted in the absence of any commercial or financial relationships that could be construed as a potential conflict of interest.

## Publisher’s note

All claims expressed in this article are solely those of the authors and do not necessarily represent those of their affiliated organizations, or those of the publisher, the editors and the reviewers. Any product that may be evaluated in this article, or claim that may be made by its manufacturer, is not guaranteed or endorsed by the publisher.

## Figures and Tables

**Table:** 

Construct	Dimensions	Items
Cognitive Engagement (CE)	*Conscious Attention (CA)*	1. I like learning more about violent video games
		2. I notice information related to violent video games.
		3. I pay a lot of attention to anything about violent video games.
		4. I keep up with things related to violent video games.
		5. Anything related to violent video games grabs my attention. 6. I can concentrate on violent video games’ content and story for a long time.
	*Absorption (AB)*	1. When I am playing violent video games, I forget everything else around me.
		2. Time flies when I am playing violent video games.
		3. When I am playing violent video games, I get carried away.
		4. When I am playing violent video games, I feel immersed.
		5. I feel happy when I am playing violent video games intensely.
Affective Engagement (AE)	*Dedication (DE)*	1. Violent video games inspires me.
		2. I am enthusiastic about playing violent video games.
		3. I am proud to play violent video games.
		4. I find violent video games to be full of meaning and purpose.
		5. I am excited when playing violent video games.
	*Enthusiasm (EN)*	1. I spend a lot of my discretionary time playing violent video games.
		2. I am heavily into playing violent video games.
		3. I am passionate about playing violent video games.
		4. I enjoy spending time playing violent video games.
		5. I try to fit playing violent video games into my schedule.

Behavioral Engagement (BE)	*Interaction (IN)*	1. In general, I like to get involved in discussions about violent video games. 2. I enjoy playing violent video games with like-minded other gamers.
		3. I like to be actively participating in discussions about violent video games.
		4. In general, I enjoy exchanging ideas on violent video games with other gamers.
		5. I often participate in activities relating to violent video games.
	*Social Connection (SC)*	1. I love playing violent video games with my friends.
		2. I enjoy playing violent video games when I am with others.
		3. Playing violent video games is more fun when other people around me play it too.
		1. Once in a while I cannot control the urge to strike another person in violent video games.
		2. When violent video games arises enough provocation, I may hit another person.
	*Physical Aggression*	3. If somebody hits me, I hit back.
		4. I get into fights a little more than the average person.
		5. If I have to resort to violence to protect my rights, I will.
		6. There are people who pushed me so far that we came to blows.
Aggressive Behavior		7*. I can think of no good reason for ever hitting a person.
		8. I have threatened people I know.
		9. I have become so mad that I have broken things.
	*Verbal Aggression*	1. I tell my friends openly when I disagree with them.
		2. I often find myself disagreeing with people.
		3. When people annoy me, I may tell them what I think of them.
		4. I cannot help getting into arguments when people disagree with me.
		5*. My friends say that I’m somewhat argumentative.
	*Anger*	1*. I flare up quickly but get over it quickly.
		2. When frustrated, I let my irritation show.
		3. I sometimes feel like a powder keg ready to explode.
		4. I am an even-tempered person.
		5. Some of my friends think I’m a hothead.
		6. Sometimes I fly off the handle for no good reason. 7. I have trouble controlling my temper.
	*Hostility*	1. I am sometimes eaten up with jealousy
		2. At times I feel I have gotten a raw deal out of life.
		3. Other people always seem to get the breaks.
		4. I wonder why sometimes I feel so bitter about things.
		5*. I know that “friends” talk about me behind my back.
		6*. I am suspicious of overly friendly strangers.
		7. I sometimes feel that people are laughing at me behind my back.
		8*. When people are especially nice, I wonder what they want.

## Appendix

Appendix A: Survey instrument.

Italicized and bold items are deleted due to low loadings.

## References

[ref1] Abbasi A. Z. (2022). Specifying, estimating and validating consumer eSports engagement composite model: a composite confirmatory approach. EuroMed J. Bus. doi: 10.1108/EMJB-04-2022-0068 [Epub ahead of print].

[ref2] Abbasi A. Z. Asif M. Hollebeek L. D. Islam J. U. Ting D. H. Rehman U. (2021a). The effects of consumer esports videogame engagement on consumption behaviors. J. Prod. Brand. Manag. 30, 1194–1211. doi: 10.1108/JPBM-04-2020-2839

[ref3] Abbasi A. Z. Azeem S. Farooq M. U. Hussain K. Ting D. H. Rehman U. (2022). Engagement in educational games and quality of life in early and middle childhood: evidence from a developing country. Curr. Psychol. doi: 10.1007/s12144-022-03558-1 [Epub ahead of print].

[ref4] Abbasi A. Z. Nisar S. Rehman U. Ting D. H. (2020). Impact of HEXACO personality factors on consumer video game engagement: a study on eSports. Front. Psychol. 11:1831. doi: 10.3389/fpsyg.2020.01831, PMID: 32849078 PMC7422731

[ref5] Abbasi A. Z. Rehman U. Afaq Z. Rafeh M. A. Hlavacs H. Mamun M. A. (2021b). Predicting video game addiction through the dimensions of consumer video game engagement: quantitative and cross-sectional study. JMIR Serious Games 9:e30310. doi: 10.2196/30310, PMID: 34842539 PMC8665386

[ref6] Abbasi A. Z. Ting D. H. Hlavacs H. (2016). “A revisit of the measurements on engagement in videogames: A new scale development.” in *International Conference on Entertainment Computing*. Springer, Cham, 247–252.

[ref7] Abbasi A. Z. Ting D. H. Hlavacs H. (2017). Engagement in games: developing an instrument to measure consumer videogame engagement and its validation. Int. J. Comput. Games Technol. 10:7363925. doi: 10.1155/2017/7363925

[ref8] Abbasi A. Z. Ting D. H. Hlavacs H. Costa L. V. Veloso A. I. (2019a). An empirical validation of consumer video game engagement: a playful-consumption experience approach. Entertain. Comput. 29, 43–55. doi: 10.1016/j.entcom.2018.12.002

[ref9] Abbasi A. Z. Ting D. H. Hlavacs H. Fayyaz M. S. Wilson B. (2019b). “Playful-consumption experience and consumer videogame engagement in the lens of SR model: An empirical study.” in *International Conference on Human-Computer Interaction*. Springer, Cham, 85–104.

[ref10] Abrahim S. Mir B. A. Suhara H. Mohamed F. A. Sato M. (2019). Structural equation modeling and confirmatory factor analysis of social media use and education. Int. J. Educ. Technol. High. Educ. 16:32. doi: 10.1186/s41239-019-0157-y

[ref11] Adachi P. J. Willoughby T. (2011). The effect of video game competition and violence on aggressive behavior: which characteristic has the greatest influence? Psychol. Violence 1, 259–274. doi: 10.1037/a0024908

[ref12] Adachi P. Willoughby T. (2016). The longitudinal association between competitive video game play and aggression among adolescents and young adults. Child Dev. 87, 1877–1892. doi: 10.1111/cdev.12556, PMID: 27346428

[ref13] Albina A. Abella C. Salvador A. Alvior F. Bato R. (2020). The level of aggression among players of non-violent and violent video games. Available at: https://www.academia.edu/

[ref14] American Psychological Association (2015). Resolution on violent video games. Available at: http://www.apa.org/about/policy/violent-video-games.aspx

[ref500] Anderson C. A. Carnagey N. L. Flanagan M. Benjamin A. J. Eubanks J. Valentine J. (2004). Violent video games: Specific effects of violent content on aggressive thoughts and behavior. Adv. Exp. Soc. Psychol. 36, 199–249., PMID: 18977956

[ref15] Anderson C. A. Sakamoto A. Gentile D. A. Ihori N. Shibuya A. Yukawa S. (2008). Longitudinal effects of violent video games on aggression in Japan and the United States. Pediatrics 122, e1067–e1072. doi: 10.1542/peds.2008-1425, PMID: 18977956

[ref16] Anderson C. A. Shibuya A. Ihori N. Swing E. L. Bushman B. J. Sakamoto A. (2010). Violent video game effects on aggression, empathy, and prosocial behavior in eastern and Western countries: a meta-analytic review. Psychol. Bull. 136, 151–173. doi: 10.1037/a0018251, PMID: 20192553

[ref17] Bassiouni D. H. Hackley C. (2014). Generation Z'children's adaptation to digital consumer culture: a critical literature review. J. Cust. Behav. 13, 113–133. doi: 10.1362/147539214X14024779483591

[ref18] Bassiouni D. H. Hackley C. (2016). Video games and young children’s evolving sense of identity: a qualitative study. Young Consum. 17, 127–142. doi: 10.1108/YC-08-2015-00551

[ref19] Becker J.-M. Klein K. Wetzels M. (2012). Hierarchical latent variable models in PLS-SEM: guidelines for using reflective-formative type models. Long Range Plan. 45, 359–394. doi: 10.1016/j.lrp.2012.10.001

[ref20] Beerthuizen M. G. Weijters G. van der Laan A. M. (2017). The release of grand theft auto V and registered juvenile crime in the Netherlands. Eur. J. Criminol. 14, 751–765. doi: 10.1177/1477370817717070

[ref21] Breuer J. Scharkow M. Quandt T. (2015). Sore losers? A reexamination of the frustration–aggression hypothesis for colocated video game play. Psychol. Pop. Media Cult. 4, 126–137. doi: 10.1037/ppm0000020

[ref22] Burkhardt J. Lenhard W. (2022). A meta-analysis on the longitudinal, age-dependent effects of violent video games on aggression. Media Psychol. 25, 499–512. doi: 10.1080/15213269.2021.1980729

[ref23] Buss A. H. Perry M. (1992). The aggression questionnaire. J. Pers. Soc. Psychol. 63, 452–459. doi: 10.1037/0022-3514.63.3.452 1403624

[ref24] Cheng M. T. She H. C. Annetta L. A. (2015). Game immersion experience: its hierarchical structure and impact on game-based science learning. J. Comput. Assist. Learn. 31, 232–253. doi: 10.1111/jcal.12066

[ref25] Creswell J. W. (2013). Research Design: Qualitative, Quantitative, and Mixed Methods Approaches. USA: Sage publications.

[ref26] Denson T. F. Dixson B. J. Tibubos A. N. Zhang E. Harmon-Jones E. Kasumovic M. M. (2020). Violent video game play, gender, and trait aggression influence subjective fighting ability, perceptions of men's toughness, and anger facial recognition. Comput. Hum. Behav. 104:106175. doi: 10.1016/j.chb.2019.106175

[ref27] Drummond A. Sauer J. D. Ferguson C. J. (2020). Do longitudinal studies support long-term relationships between aggressive game play and youth aggressive behaviour? A meta-analytic examination. R. Soc. Open Sci. 7:200373. doi: 10.1098/rsos.200373, PMID: 32874632 PMC7428266

[ref28] Drummond A. Sauer J. D. Ferguson C. J. Cannon P. R. Hall L. C. (2021). Violent and non-violent virtual reality video games: influences on affect, aggressive cognition, and aggressive behavior. Two pre-registered experiments. J. Exp. Soc. Psychol. 95:104119. doi: 10.1016/j.jesp.2021.104119

[ref29] Emre O. (2020). Effect of game addiction on reactive-proactive aggression in adolescents. Ann. Med. Res. 27, 85–91. doi: 10.5455/annalsmedres.2019.12.799

[ref30] Faul F. Erdfelder E. Lang A.-G. Buchner A. (2007). G* power 3: a flexible statistical power analysis program for the social, behavioral, and biomedical sciences. Behav. Res. Methods 39, 175–191. doi: 10.3758/BF03193146, PMID: 17695343

[ref31] Ferguson C. J. (2020). Aggressive video games research emerges from its replication crisis (sort of). Curr. Opin. Psychol. 36, 1–6. doi: 10.1016/j.copsyc.2020.01.002, PMID: 32146151

[ref32] Ferguson C. J. Copenhaver A. Markey P. (2020). Reexamining the findings of the American Psychological Association’s 2015 task force on violent media: a meta-analysis. Perspect. Psychol. Sci. 15, 1423–1443. doi: 10.1177/1745691620927666, PMID: 32777188

[ref33] Ferguson C. J. Wang J. C. (2019). Aggressive video games are not a risk factor for future aggression in youth: a longitudinal study. J. Youth Adolesc. 48, 1439–1451. doi: 10.1007/s10964-019-01069-0 31273603

[ref34] Ferguson C. J. Wang C. J. (2021). Aggressive video games are not a risk factor for mental health problems in youth: a longitudinal study. Cyberpsychol. Behav. Soc. Netw. 24, 70–73. doi: 10.1089/cyber.2020.0027 33252268

[ref35] Ge X. Ifenthaler D. (2018). “Designing engaging educational games and assessing engagement in game-based learning,” in Gamification in Education: Breakthroughs in Research and Practice. Hershey, PA: IGI Global, 1–19.

[ref36] Greitemeyer T. (2018). The spreading impact of playing violent video games on aggression. Comput. Hum. Behav. 80, 216–219. doi: 10.1016/j.chb.2017.11.022

[ref37] Greitemeyer T. Mügge D. O. (2014). Video games do affect social outcomes: a meta-analytic review of the effects of violent and prosocial video game play. Personal. Soc. Psychol. Bull. 40, 578–589. doi: 10.1177/0146167213520459, PMID: 24458215

[ref38] Griffiths R. P. Eastin M. S. Cicchirillo V. (2016). Competitive video game play: an investigation of identification and competition. Commun. Res. 43, 468–486. doi: 10.1177/0093650214565895

[ref39] Hair J. J. F. Black W. C. Babin B. J. Anderson R. E. (2010). Multivariate Data Analysis: A Global Perspective. Upper Saddle River: Pearson Education

[ref40] Hair J. F. Howard M. C. Nitzl C. (2020). Assessing measurement model quality in PLS-SEM using confirmatory composite analysis. J. Bus. Res. 109, 101–110. doi: 10.1016/j.jbusres.2019.11.069

[ref41] Hair J. F. Ringle C. M. Sarstedt M. (2011). PLS-SEM: indeed a silver bullet. J. Mark. Theory Pract. 19, 139–152. doi: 10.2753/MTP1069-6679190202

[ref42] Hair J. F. Risher J. J. Sarstedt M. Ringle C. M. (2019). When to use and how to report the results of PLS-SEM. Eur. Bus. Rev. 31, 2–24. doi: 10.1108/EBR-11-2018-0203

[ref43] Hawk C. E. Ridge R. D. (2021). Is it only the violence? The effects of violent video game content, difficulty, and competition on aggressive behavior. J. Media Psychol. 33, 134–144. doi: 10.1027/1864-1105/a000291

[ref44] Henseler J. Ringle C. M. Sarstedt M. (2015). A new criterion for assessing discriminant validity in variance-based structural equation modeling. J. Acad. Mark. Sci. 43, 115–135. doi: 10.1007/s11747-014-0403-8

[ref45] Higgins E. T. Scholer A. A. (2009). Engaging the consumer: the science and art of the value creation process. J. Consum. Psychol. 19, 100–114. doi: 10.1016/j.jcps.2009.02.002

[ref46] Hollebeek L. D. (2013). The customer engagement/value interface: an exploratory investigation. Australas. Mark. J. AMJ 21, 17–24. doi: 10.1016/j.ausmj.2012.08.006

[ref47] Hollebeek L. D. Clark M. K. Andreassen T. W. Sigurdsson V. Smith D. (2020). Virtual reality through the customer journey: framework and propositions. J. Retail. Consum. Serv. 55:102056. doi: 10.1016/j.jretconser.2020.102056

[ref48] Hollingdale J. Greitemeyer T. (2014). The effect of online violent video games on levels of aggression. PLoS One 9:e111790. doi: 10.1371/journal.pone.0111790, PMID: 25391143 PMC4229070

[ref49] Johannes N. Vuorre M. Magnusson K. Przybylski A. K. (2022). Time spent playing two online shooters has no measurable effect on aggressive affect. Collab. Psychol. 8:34606. doi: 10.1525/collabra.34606

[ref50] Kühn S. Kugler D. T. Schmalen K. Weichenberger M. Witt C. Gallinat J. (2019). Does playing violent video games cause aggression? A longitudinal intervention study. Mol. Pychiatr. 24, 1220–1234. doi: 10.1038/s41380-018-0031-7 PMC6756088 29535447

[ref51] Markey P. M. Males M. A. French J. E. Markey C. N. (2015). Lessons from Markey et al. (2015) and Bushman et al. (2015): sensationalism and integrity in media research. Hum. Commun. Res. 41, 184–203. doi: 10.1111/hcre.12057

[ref52] Parsons S. A. Malloy J. A. Parsons A. W. Burrowbridge S. C. (2012). Students’affective engagement in literacy tasks: observations of and interviews with sixth-grade students. Assoc. Literacy Educ. Res. Yearbook 34, 137–147.

[ref53] Pierre-Louis S. (2022). Essential facts about the computer and video game industry. Entertainment software association. Available at: https://www.theesa.com/resource/2022-essential-facts-about-the-video-game-industry/

[ref54] Prot S. Gentile D. A. Anderson C. A. Suzuki K. Swing E. Lim K. M. (2014). Long-term relations among prosocial-media use, empathy, and prosocial behavior. Psychol. Sci. 25, 358–368. doi: 10.1177/0956797613503854, PMID: 24335350

[ref55] Przybylski A. K. Rigby C. S. Ryan R. M. (2010). A motivational model of video game engagement. Rev. Gen. Psychol. 14, 154–166. doi: 10.1037/a0019440

[ref56] Przybylski A. K. Weinstein N. (2019). Violent video game engagement is not associated with adolescents' aggressive behaviour: evidence from a registered report. R. Soc. Open Sci. 6:171474. doi: 10.1098/rsos.171474, PMID: 30891250 PMC6408382

[ref57] Riva P. Gabbiadini A. Lauro L. J. R. Andrighetto L. Volpato C. Bushman B. J. (2017). Neuromodulation can reduce aggressive behavior elicited by violent video games. Cogn. Affect. Behav. Neurosci. 17, 452–459. doi: 10.3758/s13415-016-0490-8, PMID: 28035636

[ref58] Romanchych E. (2018). Violent video gaming, parent and child risk factors, and aggression in school-age children.

[ref59] Ruiz-Fernández A. Junco-Guerrero M. Cantón-Cortés D. (2021). Exploring the mediating effect of psychological engagement on the relationship between child-to-parent violence and violent video games. Int. J. Environ. Res. Public Health 18:2845. doi: 10.3390/ijerph18062845, PMID: 33799538 PMC8001326

[ref60] Sarstedt M. Hair J. F. Jr. Cheah J.-H. Becker J.-M. Ringle C. M. (2019). How to specify, estimate, and validate higher-order constructs in PLS-SEM. Australas. Mark. J. AMJ 27, 197–211. doi: 10.1016/j.ausmj.2019.05.003

[ref61] Schaufeli W. B. Salanova M. González-Romá V. Bakker A. B. (2002). The measurement of engagement and burnout: a two sample confirmatory factor analytic approach. J. Happiness Stud. 3, 71–92. doi: 10.1023/A:1015630930326

[ref62] Shah A. M. (2019). Honey, find me the moon: exploring engagement on dating and matrimony platforms. Young Consum. 21, 171–192. doi: 10.1108/YC-01-2019-0948

[ref63] So K. K. F. King C. Sparks B. A. Wang Y. (2016). The role of customer engagement in building consumer loyalty to tourism brands. J. Travel Res. 55, 64–78. doi: 10.1177/0047287514541008

[ref64] Tear M. J. Nielsen M. (2013). Failure to demonstrate that playing violent video games diminishes prosocial behavior. PLoS One 8:e68382. doi: 10.1371/journal.pone.0068382, PMID: 23844191 PMC3700923

[ref65] Tian Y. Gao M. Wang P. Gao F. (2020). The effects of violent video games and shyness on individuals’ aggressive behaviors. Aggress. Behav. 46, 16–24. doi: 10.1002/ab.21869, PMID: 31613405

[ref66] Triberti S. Villani D. Riva G. (2015). Moral positioning in video games and its relation with dispositional traits: the emergence of a social dimension. Comput. Hum. Behav. 50, 1–8. doi: 10.1016/j.chb.2015.03.069

[ref67] Tsang S. K. Hui E. K. Law B. (2012). Self-efficacy as a positive youth development construct: a conceptual review. Sci. World J. 2012, 1–7. doi: 10.1100/2012/452327 PMC3351108 22645423

[ref68] Vivek S. D. Beatty S. E. Dalela V. Morgan R. M. (2014). A generalized multidimensional scale for measuring customer engagement. J. Mark. Theory Pract. 22, 401–420. doi: 10.2753/MTP1069-6679220404

[ref69] Wu J. Holsapple C. (2014). Imaginal and emotional experiences in pleasure-oriented IT usage: a hedonic consumption perspective. Inf. Manag. 51, 80–92. doi: 10.1016/j.im.2013.09.003

[ref70] Zhang Q. Cao Y. Tian J. (2021a). Effects of violent video games on aggressive cognition and aggressive behavior. Cyberpsychol. Behav. Soc. Netw. 24, 5–10. doi: 10.1089/cyber.2019.0676, PMID: 33370158

[ref71] Zhang Q. Tian J. Chen L. Cao Y. (2021b). Effects of violent video games on aggressive behaviors among children: the role of anger and trait aggression in China. ResearchSquare [Preprint]. doi: 10.21203/rs.3.rs-139260/v1

